# Silencing of the Long Noncoding RNA *MYCNOS1* Suppresses Activity of *MYCN*-Amplified Retinoblastoma Without *RB1* Mutation

**DOI:** 10.1167/iovs.61.14.8

**Published:** 2020-12-03

**Authors:** Duangporn Saengwimol, Pamorn Chittavanich, Natanan Laosillapacharoen, Atthapol Srimongkol, Vijender Chaitankar, Duangnate Rojanaporn, Rangsima Aroonroch, Bhoom Suktitipat, Chonticha Saisawang, Saovaros Svasti, Suradej Hongeng, Rossukon Kaewkhaw

**Affiliations:** 1 Research Center, Faculty of Medicine, Ramathibodi Hospital, Mahidol University, Bangkok, Thailand; 2 Section of Translational Medicine, Faculty of Medicine, Ramathibodi Hospital, Mahidol University, Bangkok, Thailand; 3 Lymphocyte Cell Biology Section, Molecular Immunology and Inflammation Branch, National Institute of Arthritis and Musculoskeletal and Skin Diseases, National Institutes of Health, Bethesda, Maryland, United States; 4 Department of Ophthalmology, Faculty of Medicine, Ramathibodi Hospital, Mahidol University, Bangkok, Thailand; 5 Department of Pathology, Faculty of Medicine, Ramathibodi Hospital, Mahidol University, Bangkok, Thailand; 6 Department of Biochemistry, Faculty of Medicine, Siriraj Hospital, Mahidol University, Bangkok, Thailand; 7 Integrative Computational BioScience Center, Mahidol University, Nakhon Pathom, Thailand; 8 Institute of Molecular Biosciences, Mahidol University, Nakhon Pathom, Thailand; 9 Department of Biochemistry, Faculty of Science, Mahidol University, Bangkok, Thailand; 10 Thalassemia Research Center, Institute of Molecular Biosciences, Mahidol University, Nakhon Pathom, Thailand; 11 Department of Pediatrics, Faculty of Medicine, Ramathibodi Hospital, Mahidol University, Bangkok, Thailand

**Keywords:** retinoblastoma, *MYCN*, long noncoding RNA *MYCNOS1*, *RB1*, photoreceptor signature, drug response

## Abstract

**Purpose:**

*MYCNOS* (*MYCN* opposite strand) is co-amplified with *MYCN* in pediatric cancers, including retinoblastoma. *MYCNOS* encodes several RNA variants whose functions have not been elucidated in retinoblastoma. Thus, we attempted to identify *MYCNOS* variants in retinoblastoma and aimed to decipher the role of *MYCNOS* variant 1 (*MYCNOS1*) on the activity of *MYCN*-amplified retinoblastoma.

**Methods:**

The profiles of *MYCNOS* variants and *MYCN* status were determined in 17 retinoblastoma tissues, cell lines, retinas, and retinal organoids. A functional study of *MYCNOS1* expression was conducted in patient-derived tumor cells and in retinoblastoma cell lines via short hairpin RNA-mediated gene silencing. We carried out *MYCN* expression, cell viability, cell cycle, apoptosis, soft agar colony formation, and transwell assays to examine the role of *MYCNOS1* in *MYCN* and cell behaviors. We analyzed a transcriptome of *MYCN*-amplified retinoblastoma cells deficient for *MYCNOS1* and, finally, tested the responses of these cells to chemotherapeutic agents.

**Results:**

Expression of *MYCNOS1* was associated with the expression and copy number of *MYCN*. Knockdown of *MYCNOS1* caused instability of the *MYCN* protein, leading to cell cycle arrest and impaired proliferation and chemotaxis-directed migration in *MYCN*-amplified retinoblastoma cells in which *RB1* was intact. *MYCNOS1* expression was associated with gene signatures of photoreceptor cells and epithelial–mesenchymal transition. *MYCNOS1* silencing enhanced the response of retinoblastoma cells to topotecan but not carboplatin.

**Conclusions:**

*MYCNOS1* supports progression of retinoblastoma. Inhibition of *MYCNOS1* expression may be necessary to suppress *MYCN* activity when treating *MYCN*-amplified cancers without *RB1* mutation.

Retinoblastoma is a retinal tumor of infancy and childhood. Although the biallelic loss of *RB1* in retina cells has been known for many decades to initiate the disease,^1^^,^^2^ high focal amplification of *MYCN* has been identified as the primary driver in a novel subtype that is found in the 1% to 2% of patients whose tumors carry the wild-type *RB1* gene.^3^^–^^5^ This oncogene-driven retinoblastoma type is a very early-onset unilateral tumor that exhibits more aggression than the classical *RB1*-deficient retinoblastoma.^5^ *MYCN*-amplified retinoblastoma without *RB1* mutation appears to have histopathological and genetic characteristics similar to those of other *MYCN*-amplified embryonic tumors, such as neuroblastoma,^3^^,^^5^^,^^6^ even though the tumor features a retinoblastoma-associated gene expression profile.^7^

In oncogenic-driven retinoblastoma, *MYCN* is focally amplified with >28 copies, spanning 1 to 5 Mb and encompassing neighboring genes.^4^^,^^5^^,^^8^ *MYCNOS* (*MYCN* opposite strand) is located on the DNA strand opposite to *MYCN* with extensive head-to-head overlap; it is thus inevitably co-amplified in all cases of *MYCN*-amplified retinoblastoma.^4^^,^^5^ Additionally, the association of high *MYCNOS* transcript levels with *MYCN* amplification and expression has been widely reported in neuroblastoma.^9^^–^^13^

*MYCNOS* encodes several RNA variants that exert their functions as long noncoding RNA or coding RNA and may functionally characterize human diseases.^12^ Most studies have focused on the role of variant 2, or *MYCNOS2* (NR_161162.1), in tumorigenesis, where *MYCNOS2* is associated with poor clinical outcomes in patients with neuroblastoma.^10^^,^^12^^,^^13^ *MYCNOS2* transcripts serving as a noncoding RNA facilitate *MYCN* expression.^10^^,^^13^^,^^14^ Moreover, protein-coding *MYCNOS2* facilitates the stabilization of *MYCN* oncoprotein, activation of Wnt/β-catenin signaling, and generation of an anti-apoptotic protein, which supports metastasis, chemoresistance, and survival of cancers.^12^^,^^15^^,^^16^

However, the function of transcript variant 1, or *MYCNOS1* (NR_110230.2), has not been fully elucidated. One study reported that silencing the long noncoding RNA *MYCNOS1* results in reduced cell proliferation of *MYCN*-amplified neuroblastoma and rhabdomyosarcoma.^11^ *MYCNOS* appears to play a key role in cancer progression, but whether it acts as a silent passenger or is a pathogenic consequence of *MYCN* amplification in retinoblastoma is not known. Here, we characterize the expression profile of all five *MYCNOS* variants in human retinoblastoma tissues, cell lines, retina, and retinal organoids. Based on these observations, we hypothesize that *MYCNOS1* promotes oncogenesis and has functional relevance with *MYCN* in *MYCN*-amplified retinoblastoma.

## Materials and Methods

### Human Samples

Retinoblastoma samples were collected from enucleated globes of patients at Ramathibodi Hospital, Mahidol University (Bangkok, Thailand). Fresh surgical specimens were used for genomic DNA and total RNA extraction or directly processed for derivation of cell lines.^17^ Blood was drawn from patients, and peripheral blood mononuclear cells were isolated from blood with Ficoll-Paque PLUS reagent (GE Healthcare, Uppsala, Sweden) in accordance with the manufacturer's instructions for genomic DNA extraction. Case characteristics of the 17 patients are listed in the Table. Postmortem eye globes were collected by the Thai Red Cross Eye Bank (Bangkok, Thailand) for cornea donation. The remaining eye globes were used for dissection to extract the neural retina. The lens, iris, and vitreous were discarded, and the choroid/retinal pigment epithelium layers were removed from the retina for sample collection. All experimental protocols were approved by the institutional review board at the Faculty of Medicine, Ramathibodi Hospital, Mahidol University. All methods were performed in accordance with the relevant guidelines and regulations. Informed consent was obtained from a parent of each patient before samples were collected. Retinal organoids were generated from H9 human embryonic stem cells (ESCs) to represent fetal tissues, according to a previous protocol.^18^

**Table.** Case Characteristics of 17 Patients with Retinoblastoma

Characteristics	Cases, *n* (%)
Age at diagnosis (mo)	
<12	6 (35)
≥12	11 (65)
Sex	
Female	11 (65)
Male	6 (35)
Classification*	
Group D eye	4 (23)
Group E eye	11 (65)
Extraocular retinoblastoma	2 (12)
Pathological features**^†^**	
High risk	14 (82)
Low risk	3 (18)

* Based on the International Intraocular Retinoblastoma Classification.

† A high-risk pathological feature is defined as presentation of retrolaminar optic nerve invasion and/or massive choroidal invasion at ≥3 mm in diameter, or anterior segment and any degree of concomitant non-massive choroidal and prelaminar/laminar optic nerve invasions.

### Patient-Derived Cells and Cell Line Cultures

Patient-derived retinoblastoma cells (RB170 cells) were established using a previously reported protocol^17^ as suspension and organoid cultures. Briefly, tumor tissue was dissociated, and the resulting cells were cultured in the growth medium developed previously.^17^ A final concentration of 1% Matrigel matrix solution (Corning, Inc., Corning, NY, USA) was added in suspension culture, and that of 65% Matrigel matrix (growth factor reduced) was used to embed cells for organoid culture. RB170 was manually dissociated and passaged at a 1:2 or 1:4 ratio once a week for suspension culture or every 3 weeks for organoids. Cold freezing medium (culture medium containing 10% dimethyl sulfoxide) was used to freeze cells at −80°C for 24 hours before long-term storage in liquid nitrogen. A human retinoblastoma cell line (Y79; American Type Culture Collection, Manassas, VA, USA) was maintained in RPMI-1640 (HyClone Laboratories, Inc., Logan, UT, USA) containing 15% fetal bovine serum (Sigma-Aldrich, St. Louis, MO, USA), 100 U/mL penicillin, 100 µg/mL streptomycin, and 0.25 µg/mL amphotericin B. Medium was changed every 3 days for both RB170 and Y79 cells.

### Short Hairpin RNA-Mediated Gene Silencing

Short hairpins (Supplementary Table S1) targeting *MYCNOS1* transcripts (two independent target regions) and non-targeting short hairpin controls (sh-NC) were cloned in pZIP-hEF1-alpha-ZsGreen-Puro vectors (Transomic Technologies, Inc., Huntsville, AL, USA). The ZIP lentiviral vector (pZIP) contained a gene cassette in which human elongation factor 1 alpha promoter (hEF-1α) drove the expression of green fluorescent marker (ZsGreen), puromycin-resistant gene, and UltramiR scaffold (Transomic)-loaded short hairpin RNA (shRNA). An element for internal ribosome entry sites was inserted between the fluorescent marker and puromycin-resistant gene. Lentivirus was produced by transfecting 293T cells with shRNA plasmids and helper plasmids pMDLg/pRRE, pRSV-Rev, and pMD2.G (12251, 12253, and 12259; Addgene, Watertown, MA, USA) using X-tremeGENE HP transfection reagent (Roche, Mannheim, Germany). Viral supernatant was collected 48 and 72 hours after transfection, filtered through a 0.45-µm filter, and concentrated using Lenti-X Concentrator (Takara Bio USA, Inc., Mountain View, CA, USA) in accordance with the manufacturer's instructions. The multiplicity of infection (MOI) was determined, and an MOI of 3 with 4-µg/mL polybrene was used to transfect 5 × 10^5^ cells. Cells were cultured for 72 hours before stable cell lines were selected with 0.4-µg/mL puromycin. The purity of ZsGreen-positive cells was confirmed by flow cytometry after selection.

Methods for genomic analysis, RNA expression analysis, western blotting, histology, immunofluorescence and imaging, live cell imaging, RNA sequencing, soft agar colony formation, cell viability, drug testing, and migration and cell cycle assays are listed in the Supplementary Materials. Data availability is also listed in the Supplementary Materials.

## Results

### Expression of *MYCNOS* Variants in Retinoblastoma

**Figure 1.**
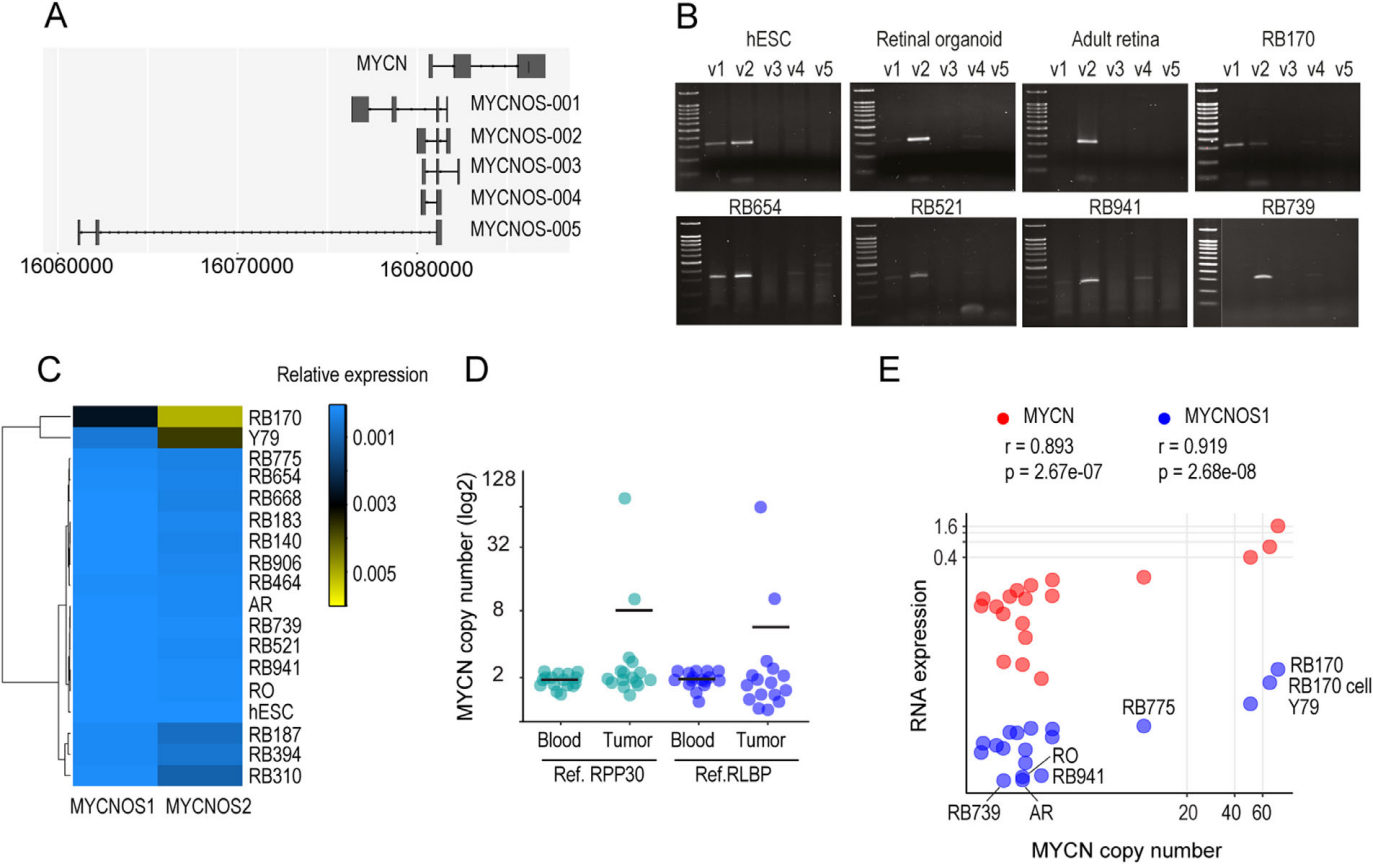
Differentially expressed *MYCNOS* RNA and association of *MYCN* copy number with *MYCNOS1* and *MYCN* expression. (**A**) Schematic representation of five RNA variants of *MYCNOS* and *MYCN* RNA (EnsDb.Hsapiens.v75-based gene model). (**B**) Expression levels of *MYCNOS* variants by RT-PCR. (**C**) Heatmap showing expression levels of *MYCNOS1* and *MYCNOS2* by quantitative RT-PCR. RNA was extracted from retinoblastoma tissues (RB), the Y79 cell line, human embryonic stem cell (hESC), hESC-derived retinal organoid (RO, representative of fetal retina), and adult retina (AR). The results for RB417, RB214, and RB581 are absent due to a limitation of RNA materials. (**D**) *MYCN* copy number of 17 tumor and paired blood DNA by droplet digital PCR. (**E**) Correlations of *MYCN* copy number with expression of *MYCN* and *MYCNOS1* examined in tumor and retinal tissues, patient-derived retinoblastoma cells (RB170 cells), and Y79. Correlation analysis was conducted using the Pearson correlation method in which the correlation coefficient (*r*) was computed. For *P* < 0.05, two variables were significantly correlated.

Expression levels of all five *MYCNOS* variants were examined in retinoblastoma tissues compared with adult retina, retinal organoids, and the Y79 retinoblastoma cell line (Figs. 1A, 1B). We found that *MYCNOS1* and *MYCNOS2* were expressed in tumors (Figs. 1B, 1C). Although *MYCNOS2* was detected in all tumors and normal tissues, *MYCNOS1* was rarely expressed in adult retina and retinal organoids (Fig. 1C; Supplementary Figs. S1A–S1C). RB739 and RB941 retinoblastoma tissues showed expression levels of *MYCNOS1* and *MYCNOS2* similar to those of normal tissues, suggesting possible contamination of normal retina (Fig. 1C). However, *MYCNOS1* was highly expressed in RB170 tissue and Y79 (Fig. 1C; Supplementary Figs. S1A–S1C). Expression of *MYCNOS1* was downregulated in human ESC-derived retinal organoids representative of fetal retina^18^^,^^19^ compared with ESCs, suggesting that its downregulation was required for retinogenesis, whereas high expression levels are implicated in tumorigenesis (Supplementary Figs. S1A, S1B).

### Copy Number and Expression of *MYCN* and Association with *MYCNOS1*

We then examined *MYCN* status regarding gene copy number and expression in these tumor tissues. Droplet digital PCR indicated that RB170 tissue carried high copy numbers of *MYCN*: 90 and 75 based on *RPP30* and *RLBP* reference genes, respectively (Fig. 1D). Ten copies of *MYCN* were detected in RB775, considered to be low *MYCN* amplification,^5^ whereas other tumor tissues carried about two copies compared with blood DNA (Fig. 1D). As expected, *MYCN* expression levels were relatively high in *MYCN*-amplified tumors (Fig. 1E; Supplementary Figs. S1D, S1E). The copy number of *MYCN* was positively correlated with the expression of *MYCN* and *MYCNOS1* (Fig. 1E). Additionally, *MYCN* expression was correlated with *MYCNOS1* expression (Supplementary Fig. S1C). The expression profile of *MYCNOS1* implied its oncogenic role, particularly in tumor cells with high *MYCN* amplification, and suggested functional relevance of *MYCN*.

### *MYCN* Amplification without *RB1* Mutation in RB170 Retinoblastoma

Whole-genome analysis of RB170 tumor tissue and matched germline DNA revealed high *MYCN* amplification (75 copies) (Supplementary Fig. S1F). *MYCN* amplicons spanned 1.1 Mb, encompassing *MYCNOS*, *LINC01804*, *MYCNUT*, *GACTA3*, *NBAS*, and *DDX1*, consistent with the amplicon size by whole-genome SNP array (Supplementary Fig. S1F and not shown). Somatic mutations were rarely detected (Supplementary Fig. S1G). No alterations of the *RB1* sequence were detected in tumor or blood DNA samples, consistent with the results of Sanger sequencing and multiplex ligation-dependent probe amplification (MLPA; not shown). Additionally, the sequence of *RB1* mRNA from tumors was not changed (Supplementary Fig. S1H). Molecular testing confirmed that RB170 tumor tissue had a wild-type copy of *RB1*, even though there was evidence of loss of heterozygosity spanning *RB1* detected only in the tumor (47 Mb). Histologically, rosettes were absent in tumor tissue, and tumor cells had round nuclei with prominent large nucleoli, which were readily distinguishable from the classical type of retinoblastoma (Supplementary Figs. S1I–S1K). Age at diagnosis was 3 months, and the tumor volume was large. Molecular, histological, and clinical features indicated that RB170 could be classified as a new subtype in which the disease is driven by high *MYCN* amplification.^5^

### Characterization of Tumor Cells Derived from *MYCN*-Amplified RB170 Tumor Tissue

**Figure 2.**
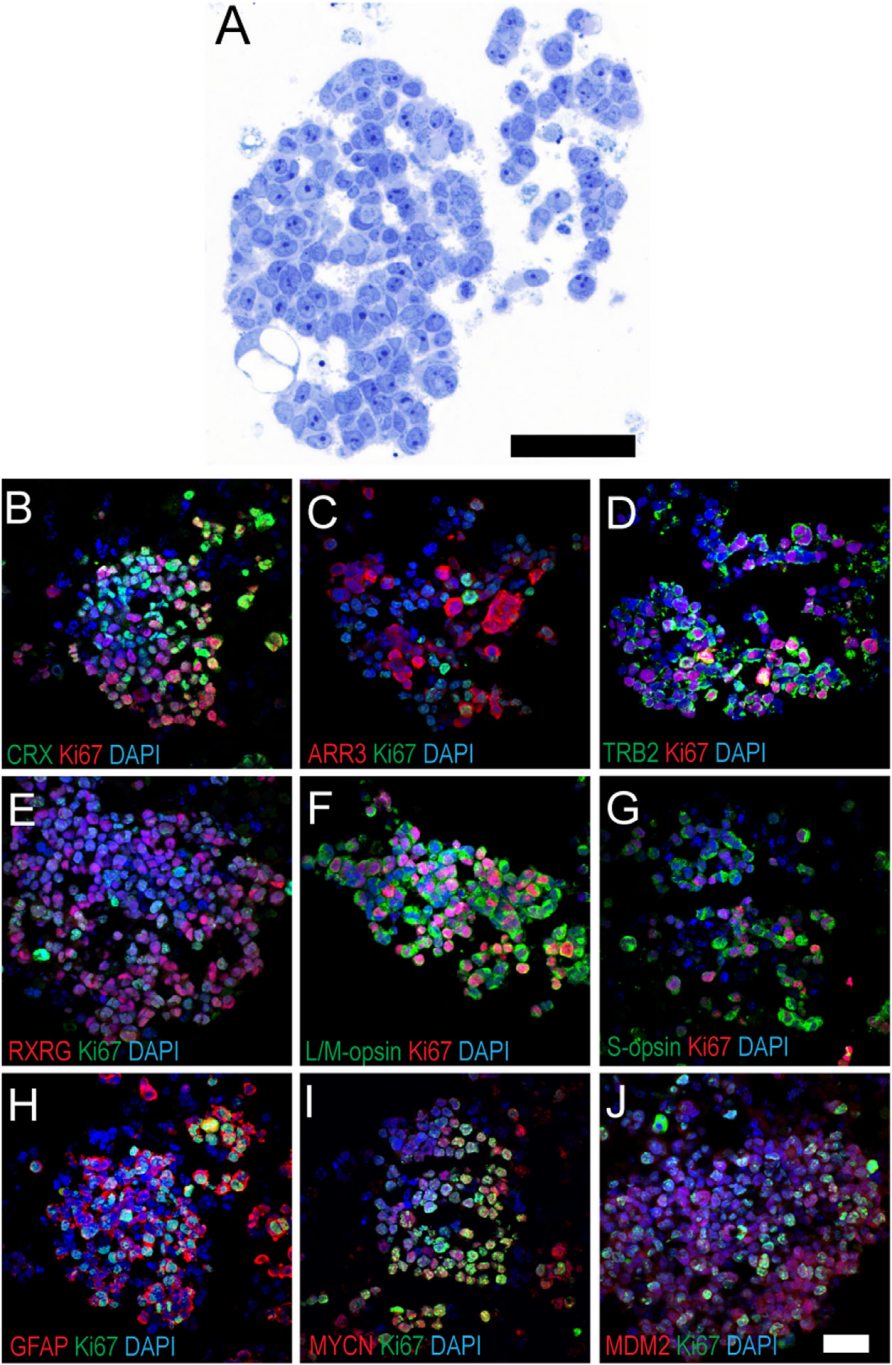
RB170 tumor cells derived from *MYCN*-amplified retinoblastoma without *RB1* mutation maintain cone-specific signaling circuitry. (**A**) Section of RB170 showing large tumor cells with prominent nucleoli, corresponding to cells in the parental tissue (Supplementary Fig. S1I). (**B**–**J**) Immunostaining of RB170 tumor cultures for CRX (**B**), ARR3 (**C**), TRβ2 (**D**), RXRG (**E**), L/M opsin (**F**), S opsin (**G**), GFAP (**H**), MYCN (**I**), and MDM2 (**J**), co-stained with Ki67 indicative of neoplastic cells. See Supplementary Fig. S2 for negative staining for other markers. *Scale bar*: 50 µm.

We generated RB170 cells grown in organoid or cell suspension cultures from *MYCN*-amplified retinoblastoma without mutations in *RB1*.^17^ Tumor cells were large and had prominent nucleoli, corresponding to the tumor cells in parental tissue (Fig. 2A; Supplementary Fig. S1I). RB170 tumor cells had cone properties, as shown by CRX and ARR3 expression (Figs. 2B, 2C). M/L (RXRγ, TRβ2, and M/L opsin) and S (S-opsin) cone-specific proteins were expressed, and glial fibrillary acidic protein (GFAP), indicative of glial cells, was also detected (Figs. 2D−2H). Nevertheless, proteins associated with other retinal cell types, including rod, retinal ganglion, interneuron, and bipolar cells, were not detected in RB170 cells (Supplementary Figs. S2A–S2F). Ki67 staining showed that tumor cultures were comprised of non-proliferative and proliferative cells (Figs. 2B–2H). Interestingly, cells staining positively for retinal markers co-expressed Ki67, suggesting that the proliferative cells had cone (M/L and S) and glial characteristics (Figs. 2B–2H). The proliferative cells of RB170 differed from those of *RB1*-deficient retinoblastoma, where M/L opsin is primarily expressed.^17^^,^^20^ Additionally, *MYCN* and MDM2 oncoproteins, which constitute a cone-signal circuitry, were detected and co-expressed with Ki67 (Figs. 2I, 2J).

RB170 cells maintained *MYCNOS1* expression, *MYCN* amplification (67 copies), and *MYCN* expression at high levels while retaining *RB1* status, as identified in the tissue by sequencing and MLPA (Fig. 1E). The Y79 cell line, known to be *RB1* deficient, carried 50 copies of *MYCN* and expressed *MYCN* and *MYCNOS1* (Fig. 1E). We then determined the function of *MYCNOS1* in RB170 and Y79 tumor cells.

### Effect of *MYCNOS1* Knockdown on *MYCN* in *MYCN*-Amplified Retinoblastoma Cells

**Figure 3.**
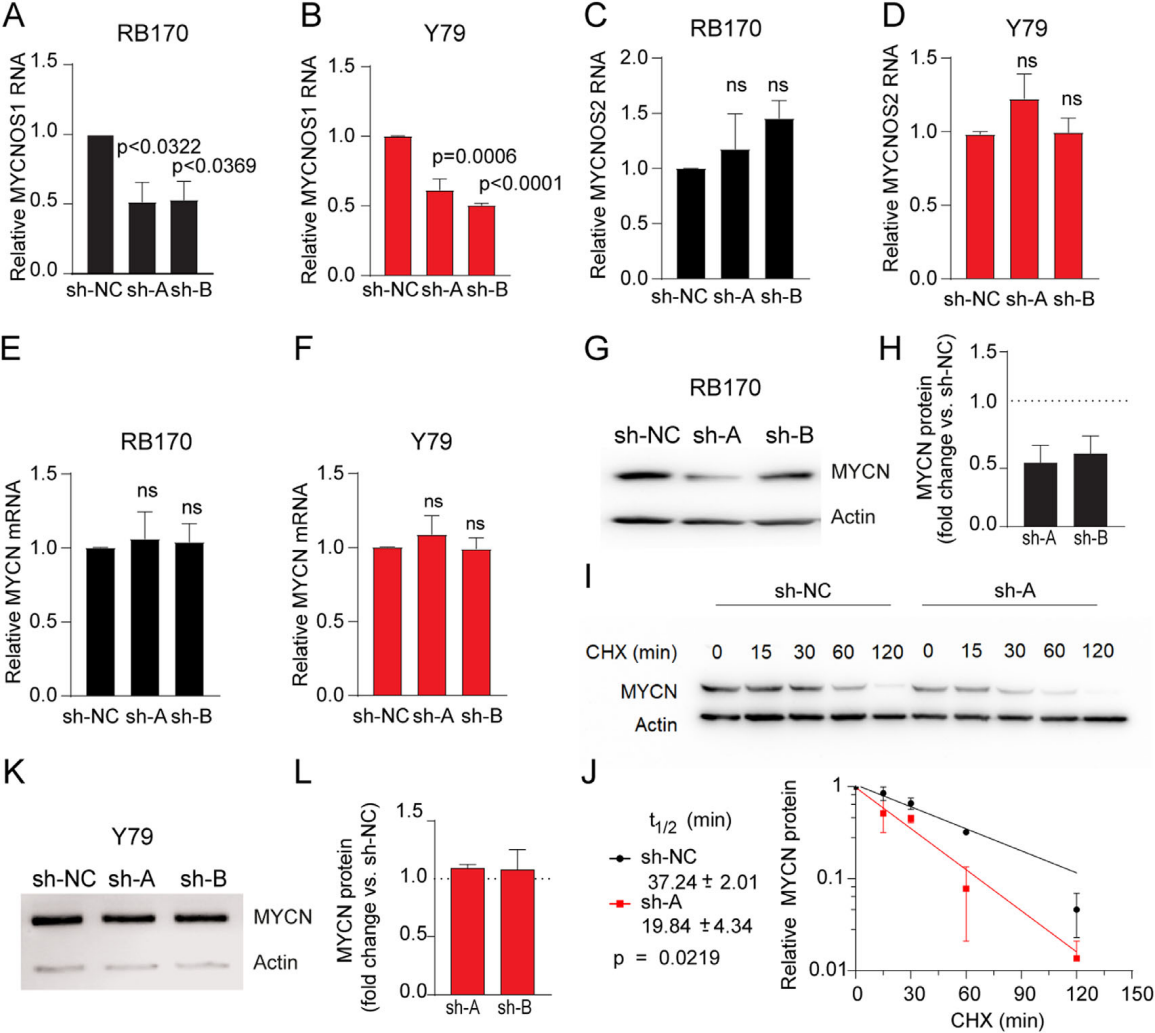
*MYCNOS1* silencing reduces stability of *MYCN* protein in *MYCN*-amplified retinoblastoma without *RB1* mutation. (**A**, **B**) *MYCNOS1* expression following *MYCNOS1* silencing (sh-A and sh-B vs. a non-targeting control, sh-NC) in *MYCN*-amplified retinoblastoma RB170 (**A**) and Y79 (**B**) by quantitative RT-PCR. (**C**–**F**) *MYCNOS2* (**C**, **D**) and *MYCN* (**E**, **F**) transcript levels following *MYCNOS1* silencing in RB170 and Y79 by quantitative RT-PCR. (**G**–**L**) *MYCN* protein levels and protein stability assay in RB170 (**G**–**J**) and *MYCN* protein levels in Y79 (**K**, **L**) following *MYCNOS1* silencing by western blotting of whole lysate and densitometry. Cycloheximide (CHX) was used to inhibit protein synthesis. Means ± SEM are shown, and at least three experiments were conducted independently. One-way ANOVA followed by Tukey's multiple comparison test and Student's *t*-test were used to measure the significance of RNA and protein levels and protein half-lives, respectively. For *P* < 0.05, the results were concluded to be statistically significant; ns, not significant.

Stably expressed shRNA targeting *MYCNOS1* (sh-A and sh-B) successfully reduced the levels of *MYCNOS1* RNA compared with sh-NC in *MYCN*-amplified RB170 and Y79 cells (Figs. 3A, 3B). *MYCNOS2* expression was not altered following *MYCNOS1* depletion, indicating a lack of compensation for the loss of variant 1 (Figs. 3C, 3D). We found that the levels of *MYCN* mRNA were not affected in RB170 and Y79 deficient for *MYCNOS1* (Figs. 3E, 3F). However, levels of *MYCN* protein were decreased following *MYCNOS1* depletion in RB170 (Figs. 3G, 3H). Concurrently, the half-life of *MYCN* protein was reduced (19.84 ± 4.34 vs. 37.24 ± 2.01 minutes; *P* = 0.0219), which confirmed that *MYCNOS1* modulated *MYCN* posttranscriptionally (Figs. 3I, 3J). In contrast, *MYCN* protein levels were not altered in Y79 cells deficient in *MYCNOS1*; *RB1* protein was completely absent in Y79, which might contribute to the levels of *MYCN* after *MYCNOS1* silencing (Figs. 3K, 3L). Regarding *MYCN* protein levels, the results suggest that knocking down *MYCNOS1* mainly affected *MYCN*-amplified tumor cells without the *RB1* mutation.

### Activity of *MYCN*-Amplified Retinoblastoma Cells Following *MYCNOS1* Knockdown

**Figure 4.**
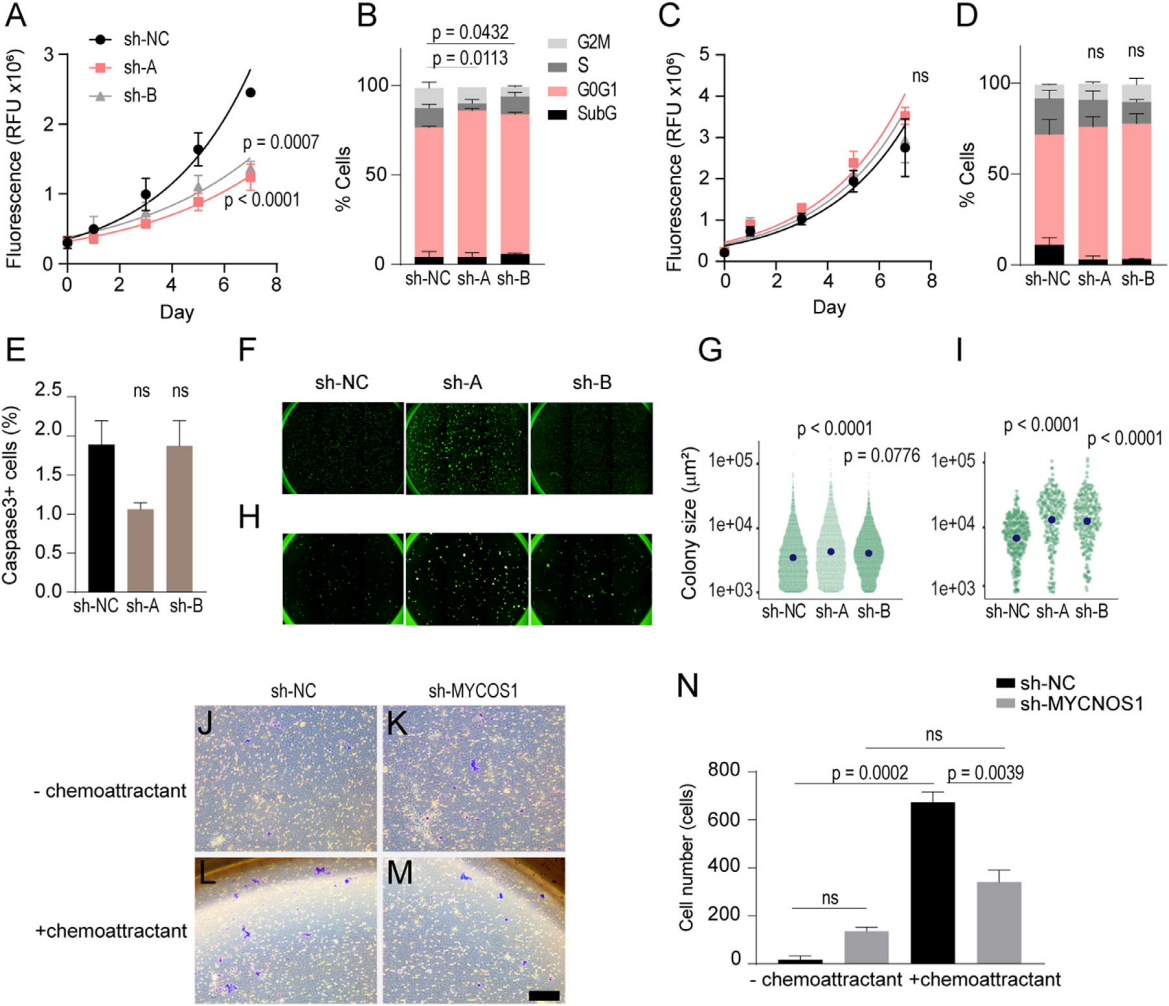
*MYCNOS1* silencing suppresses cell proliferation and migration. (**A**–**D**) Viability assay measuring proliferative rate and cell cycle analysis of RB170 (**A**, **B**) and Y79 (**C**, **D**). (**E**) Immunostaining for caspase 3, an apoptotic marker, of RB170. (**F**–**I**) Soft agar colony formation assay of RB170 (**F**, **G**) and Y79 (**H**, **I**). Each point on the scatterplots represents individual colonies (see other initial seeding density in Supplementary Fig. S3). (**J**–**N**) Chemotaxis-directed migration of RB170 in transwell assays (**J**–**M**) and the number of transmigrating cells (**N**). Means ± SEM are shown, and three experiments were conducted independently. One-way ANOVA followed by Tukey's multiple comparison test was used to test the differences between groups. For *P* < 0.05, the results were concluded to be statistically significant; ns, not significant. *Scale bar*: 200 µm.

The cell viability assay indicated a lower proliferative rate in RB170 deficient for *MYCNOS1* (Fig. 4A). This concurred with G_1_/S cell cycle arrest, which supported the role of *MYCN* protein when decreased, leading to cell arrest (Fig. 4B). Cell proliferation and cell cycle, however, were not altered following *MYCNOS1* knockdown in the Y79 cell line, which appeared to divide more rapidly (2.6 days; 95% confidence interval [CI], 2.2–3.5) than RB170 (3.7 days; 95% CI, 3.0–4.6) (Figs. 4C, 4D). The number of cells staining positive for the apoptotic marker caspase-3 was not altered after *MYCNOS1* knockdown in RB170 (Fig. 4E). The soft agar colony formation assay showed that *MYCNOS1*-deficient tumor cells formed larger colonies (Figs. 4F–4I; Supplementary Fig. S3). Colony areas of 6865.0 ± 153.0 µm^2^ and 5442.0 ± 63.6 µm^2^ were measured for RB170 cells with sh-A and sh-B compared with that of 5171 ± 89.3 µm^2^ for sh-NC-RB170 (mean ± SEM; *P* < 0.0001 and *P* = 0.0776, respectively) (Figs. 4F, 4G). This was consistent with Y79: 19250.0 ± 1028.0 µm^2^ and 17731.0 ± 1025.0 µm^2^ for sh-A and sh-B vs. 8689.0 ± 336.0 µm^2^ for sh-NC (*P* < 0.0001 and *P* = 0.0001, respectively) (Figs. 4H, 4I). The numbers of colonies of both cell lines deficient in *MYCNOS1* did not differ statistically from that of cells with sh-NC (Figs. 4F–4I).

Because proliferation of *MYCNOS1*-deficient RB170 was impaired, a large colony was not solely dependent on cell proliferation (Figs. 4A, 4B, 4F, 4G). Measurement of cell velocity magnitude from live cell imaging indicated that *MYCNOS1*-deficient RB170 had increased motility, with faster two-dimensional (2D) speed (5.75 ± 0.43 µm/h and 5.13 ± 1.41 µm/h for sh-A and sh-B vs. 4.09 ± 0.55 µm/h for sh-NC; *P* = 0.0284 and *P* = 0.2142, respectively) (Supplementary Figs. S4A–S4D). *MYCNOS1*-depleted RB170 formed dendrites and exhibited increased motility, moving toward or away from others (Supplementary Fig. S4E; Supplementary Video). This increased motility of retinoblastoma cells deficient in *MYCNOS1* could contribute to the movement of cells through agarose gel^21^ to form aggregates with contacting cells, resulting in large colonies.

We questioned, however, whether this 2D speed was related to chemotaxis-directed migration, which is involved in tumor dissemination, given that this RB170 patient had orbital retinoblastoma and brain metastasis. We then performed a transwell-based, three-dimensional migration assay.^22^ Tumor cells were greatly dependent on chemoattractant for migration (16 ± 16 cells without chemoattractant vs. 673 ± 44 cells with chemoattractant for sh-NC; *P* = 0.0002), and *MYCNOS1* silencing resulted in decreased transmigration of tumor cells (341 ± 50 cells vs. 673 ± 44 cells for sh-NC; *P* = 0.0039) (Figs. 4J–4N). Indeed, knocking down *MYCNOS1* impaired chemotaxis-directed migrating ability, given that migration of *MYCNOS1*-deficient RB170 cells was independent of chemoattractant (Figs. 4K, 4M, 4N). This indicated that *MYCNOS1* positively controlled migration and that increased 2D speed of *MYCNOS1*-depleted RB170 was related to cellular processes other than chemotaxis-directed tumor migration.

### *MYCNOS1*-Mediated Regulation of Photoreceptor and Epithelial-Mesenchymal Transition Gene Signatures

**Figure 5.**
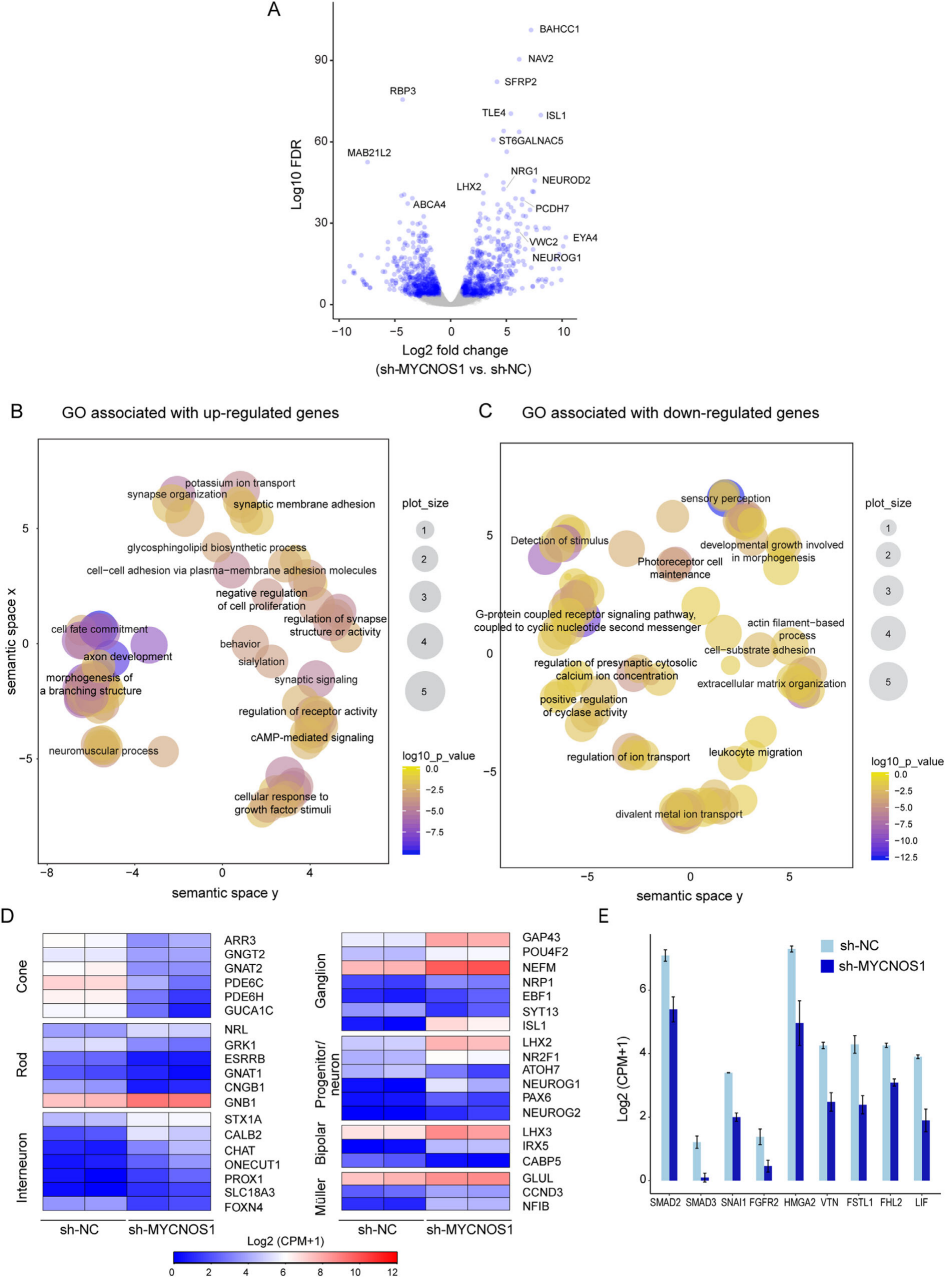
Gene signatures of photoreceptor and epithelial–mesenchymal transition were depleted in *MYCN*-amplified retinoblastoma cells deficient for *MYCNOS1*. (**A**) Volcano plot shows differentially expressed genes (*blue dots*, fold change ≥ 2 and false discovery rate [FDR]-adjusted *P* ≤ 0.01) in RB170 following *MYCNOS1* silencing. (**B**, **C**) Gene ontology terms enriched for upregulated (**B**) and downregulated (**C**) gene sets shown in the 2D space generated by applying multidimensional scaling to a matrix of the semantic similarities of the GO terms. Similar GO terms are grouped together or are closer in the plots, and cluster representatives are selected based on *P* values and dispensability scores. Bubble color indicates the *P* value; size indicates the frequency of the GO term in the underlying GO annotation database (larger bubbles indicate more general terms). (**D**) The expression of differentially expressed retinal-associated genes following *MYCNOS1* silencing. (**E**) Genes associated with EMT were significantly downregulated in *MYCNOS1*-deficient RB170 (FDR-adjusted *P* < 0.01). See Supplementary Fig. S5 for differentially expressed genes associated with EMT.

A total of 1599 genes were differentially expressed in RB170 deficient for *MYCNOS1*, with 750 being upregulated and 849 being downregulated (Fig. 5A). *BAHCC1*, *NAV2*, *SFRP2*, *RBP3*, *TLE4*, and *ISL1* were the top differentially expressed genes (Fig. 5A). Gene ontology (GO) analysis showed that cell fate commitment and axon development were the most significantly enriched processes in the upregulated gene set, and the associated genes encoded transcription factors directing the differentiation of postsynaptic neurons in retina (*ISL1*, *NEUROG1*, *NEUROD2*, *SIX1*, *PAX6*, and *PROX1*) (Figs. 5A, 5B). Additionally, expression of axon guidance molecules SLIT2, SLIT*3*, and SEMA6A was upregulated and included in GO terms of axon development and morphogenesis of a branching structure (Fig. 5B). These molecules, which can be repulsive or attractive for growing axons and migrating neurons, are implicated in neural differentiation-related cell motility (Supplementary Video).

In contrast, the expression of photoreceptor-associated genes (*RPB3* and *ABC4*) was greatly downregulated (Fig. 5A), concurrent with the enriched GO term of photoreceptor cell maintenance and several terms relating to the function of photoreceptors (Fig. 5C). We then examined the expression of retinal-associated genes within a set of differentially expressed genes following *MYCNOS1* silencing and found that expression of cone genes, including *ARR3*, *GNAT*, and *PDE6C*, was downregulated in *MYCNOS1*-depleted tumor cells (Fig. 5D). Several rod photoreceptor-specific genes whose expression is known to be depleted in retinoblastoma were downregulated, but not *NRL* and *GNB1* (Fig. 5D). Interestingly, the expression of genes associated with other retinal cell types, including interneurons, retinal ganglia, and progenitors, was upregulated (Fig. 5D). Because of the absence of cell death, a gene signature change appeared to stem from a cell fate switch after *MYCNOS* knockdown (Fig. 4E). Together, these results suggest that *MYCNOS1* maintained the cone signature in *MYCN*-amplified retinoblastoma without *RB1* mutation.

Cell–cell adhesion molecules via plasma-membrane adhesion molecules were the next enriched GO terms for upregulated genes (Fig. 5B). *CDH11*, for example, was upregulated and is a candidate tumor suppressor gene whose expression is frequently lost in advanced retinoblastoma.^23^ In contrast, cell-substrate adhesion, actin filament-based process, and extracellular matrix organization were associated with downregulated genes. These terms suggest a role of *MYCNOS1* in migration and invasiveness (Fig. 5C).

We then examined the expression of genes associated with the epithelial–mesenchymal transition (EMT). Of 1011 genes in the EMT gene database dbEMT2,^24^ we found 112 genes that were differentially expressed in our dataset, with 49 genes downregulated and 63 genes upregulated in RB170 deficient for *MYCNOS1* (Supplementary Fig. S5A). GO analysis indicated that downregulated genes were associated with the EMT term (Fig. 5E; Supplementary Fig. S5B). Additionally, positive regulation of catenin import into nucleus, SMAD protein complex assembly, and SMAD signal transduction were included in significant GO terms for downregulated genes (Supplementary Fig. S5B). Concordantly, GO terms indicative of EMT inhibition were enriched for upregulated genes, including negative regulations of canonical Wnt signaling pathway and TGF-β1 production (Supplementary Fig. S5C). Furthermore, *SFRP2* and *TLE4* (an inhibitor of Wnt/β-catenin signaling) were among the top five upregulated genes. In summary, GO analysis indicated that *MYCNOS1* controls photoreceptor signature and the EMT process.

### Sensitivity of *MYCN*-Amplified Retinoblastoma Deficient for *MYCNOS1* to Chemotherapeutic Agents

**Figure 6.**
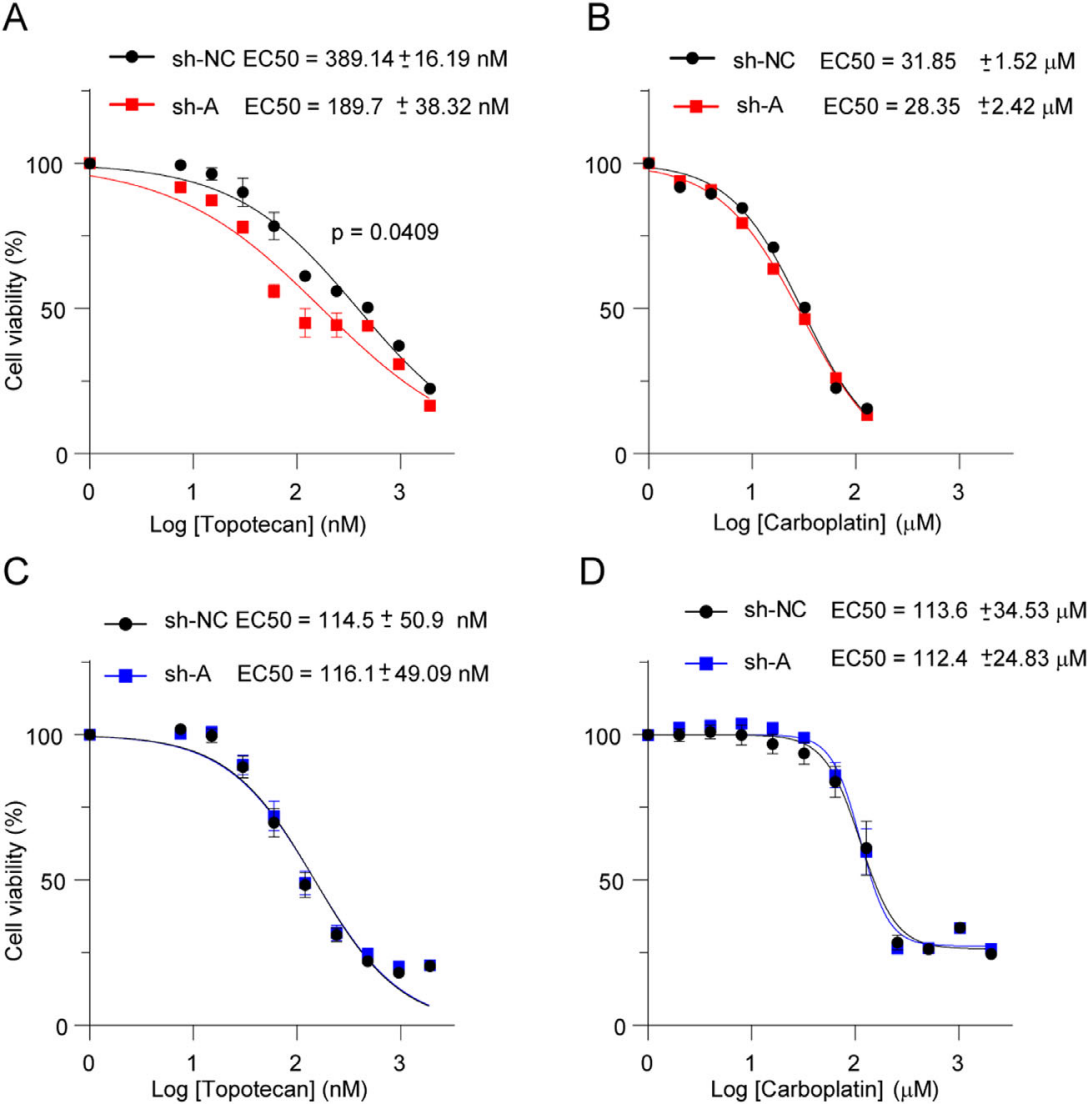
*MYCN*-amplified retinoblastoma cells deficient for *MYCNOS1* exhibited different responses to chemotherapeutic drugs. (**A**–**D**) Response of RB170 (**A**, **B**) and Y79 (**C**, **D**) cells to topotecan (**A**, **C**) and carboplatin (**B**, **D**). Means ± SEM are shown, and three experiments were conducted independently. Student's *t*-test was used to assess the significance of drug response. For *P* < 0.05, the results were concluded to be statistically significant.

Topotecan, a cell cycle–specific drug, is frequently used in intravitreal or intra-arterial chemotherapy for refractory intraocular retinoblastoma, whereas carboplatin, a non-specific cell cycle drug, is commonly used in a standard regimen for chemoreduction and is administrated intravenously. We found that *MYCNOS1* knockdown sensitized RB170 to topotecan for death, indicated by a twofold decrease in the half-maximal effective concentration (EC_50_; 389.14 ± 16.19 nM vs. 189.7 ± 38.32 nM, *P* = 0.0409), but did not affect the response of carboplatin-treated cells (Figs. 6A, 6B). This suggests that *MYCNOS1* knockdown enhanced the response of *MYCN*-amplified retinoblastoma without *RB1* mutation to a cell cycle–specific drug compared with a non-specific cell cycle drug. However, *MYCNOS1* knockdown did not alter the response of Y79 with *RB1* null mutation to topotecan or carboplatin (Figs. 6C, 6D). *RB1* was significantly upregulated in RB170 deficient for *MYCNOS1*, suggesting that *RB1* status might contribute to the different response against topotecan^25^^,^^26^ in RB170 and Y79 (Supplementary Fig. S5A). In agreement with this, the topotecan EC_50_ of RB170 was significantly higher than that of Y79 or *RB1*-deficient retinoblastoma cells without *MYCN* amplification (RB654),^17^ suggesting lower sensitivity of *MYCN*-amplified retinoblastoma with intact *RB1* to topotecan (for Y79, 389.14 ± 16.19 nM vs. 114.5 ± 50.9 nM, *P* = 0.0167; for RB654, 79.81 ± 5.31 nM, *P* = 0.0100). Nevertheless, *MYCNOS1* silencing made RB170 cells responsive to topotecan.

## Discussion

*MYCN* amplification initiates an aggressive form of retinoblastoma and co-amplifies *MYCNOS*. We showed that *MYCNOS*, encoding a long noncoding RNA or variant 1, is an oncogenic driver that promotes cell proliferation and migration, partly by regulating levels of *MYCN* protein. Furthermore, *MYCNOS1* governs the expression of genes associated with photoreceptor and EMT. The gene signature suggests a role of *MYCNOS1* in tumor differentiation and progression, which may contribute to the drug response of this oncogenic-driven retinoblastoma.

Our findings and those of others consistently show that *MYCNOS1* transcripts are highly expressed in *MYCN*-amplified tumor cells compared with non-*MYCN*-amplified cells.^11^ *MYCNOS1* transcripts regulate *MYCN* post-transcriptionally, affecting the levels of *MYCN* protein,^11^ whereas regulation at the transcriptional or posttranscriptional levels has been described for *MYCNOS2* in controlling *MYCN* expression in neuroblastoma.^10^^,^^12^^,^^13^ *MYCN* promotes cell growth and proliferation by facilitating the G_1_/S phase transition; G_1_-phase arrest is indicative of *MYCN* depletion^27^^,^^28^ and occurred in *MYCNOS1*-deficient RB170. How *MYCNOS1* positively controls *MYCN* protein stability is undetermined and requires further investigation.

*MYCNOS1* expression modulated photoreceptor and EMT signatures, which suggests its role in cancer differentiation and progression, adding to the existing body of knowledge about *MYCNOS*. Human retinoblastoma is primarily comprised of cone-like cells and has been recognized as being associated with high or low expression of cone genes.^7^^,^^17^^,^^20^^,^^29^^,^^30^ *MYCN*-driven retinoblastoma is in the group characterized by low expression of cone-associated genes,^7^ and expression of cone-associated genes was further downregulated following *MYCNOS1* depletion. This change in expression of cone genes is not a result of differential death; rather, it could result from a switch in cell fate in tumors deficient for *MYCNOS1* (Figs. 4E, 5D). Tumors with reduced cone signature express neuron- or progenitor-associated genes^31^ and respond better to chemotherapeutic agents.^7^ But, why then do *MYCNOS1*-deficient cells respond to topotecan but not carboplatin? Increased *MYCN* instability and upregulation of cyclin-dependent kinase inhibitor p27 (CDKN1B) (Supplementary Fig. S5A), an antagonist of *MYCN*,^32^ could induce cell arrest in G_1_ phase, which would enhance sensitivity to topotecan, a topoisomerase I inhibitor targeting S- and G_1_-phase cells.^33^ Loss of a cone gene signature is suggestive of tumor progression, involving enriched expression of progenitor or neuronal genes.^29^ However, it is unlikely that cells deficient for *MYCNOS1* represent advanced tumor cells, because they exhibit reduced aggressive behaviors. We hypothesize that *MYCNOS1* maintains the features of retinoblastoma/cone signature or “stemness” previously reported for *MYCNOS2*^34^ and that loss of *MYCNOS1* expression induces neuronal differentiation in *MYCN*-amplified retinoblastoma without *RB1* mutation.

EMT allows for migration, invasion, and metastasis of cancer cells and is associated with optic nerve invasion, nodal or distal metastasis, and recurrence of retinoblastoma.^35^^,^^36^ Given that this RB170 patient had orbital retinoblastoma and brain metastasis, EMT may contribute to progression of the RB170 tumor. EMT is partly achieved through activation of the SNAI1 transcription factor, which has a major role in suppressing E‐cadherin transcription.^37^ Expression of SNAI1 is induced by SMAD and HMGA2 proteins via the TGF-β/SMAD pathway in mammary epithelial cells.^38^^,^^39^ The expression of EMT-related genes was downregulated in retinoblastoma deficient for *MYCNOS1* (Fig. 5E), consistent with decreased migration ability. Upregulation of *SFRP2* and *TLE4* suggests transcriptional inactivation of Wnt/β-catenin signaling, which leads to the suppression of cancer stemness and EMT^40^^–^^42^ (Fig. 5A). *MYCNOS1* is thereby implicated in regulation of EMT^43^ and appears to have functional similarity to *MYCNOS2* protein, which promotes expression of EMT-related genes via the Wnt/β-catenin signaling pathway.^12^^,^^16^

Our findings indicate that *MYCNOS* coding for *MYCNOS1* has a pathogenic consequence of *MYCN* amplification in *MYCN*-driven retinoblastoma. In co-amplification with *MYCN*, *MYCNOS* may uniquely function only in *MYCN*-amplified tumors for cancer progression. Simultaneous expression of *MYCNOS* with *MYCN* may be needed to imitate human *MYCN*-driven retinoblastoma without the loss of *RB1* in generating a mouse model.^44^ Our findings suggest that *MYCNOS1* knockdown is a potential therapeutic strategy for *MYCN*-amplified retinoblastoma without *RB1* mutation.

A limitation of this study is the small sample size and that the sample included extremely rare cases of *MYCN*-amplified retinoblastoma without *RB1* mutation. A study with more retinoblastoma samples and additional patient-derived cell lines is necessary to strengthen our findings.

## Supplementary Material

Supplement 1

Supplement 2

## Supplementary Material

**Supplementary Video**. Time-lapse video microscopy over the course of 120 hours with images captured every 3 hours.
