# Supplementary material for: Silencing of the Long Noncoding RNA MYCNOS1 Suppresses Activity of MYCN-Amplified Retinoblastoma Without RB1 Mutation
Source: Invest Ophthalmol Vis Sci. 2020 Dec 3;61(14):8. doi: 10.1167/iovs.61.14.8 (PMC7718827; doi:10.1167/iovs.61.14.8)
Supplement: Supplement 2 [file iovs-61-14-8_s002.pdf]

Silencing of the Long Noncoding RNA *MYCNOS1* Suppresses Activity of *MYCN*-Amplified Retinoblastoma Without *RB1* Mutation

Duangporn Saengwimol,[1] Pamorn Chittavanich,[2] Natanan Laosillapacharoen,[2] Atthapol Srimongkol,[1] Vijender Chaitankar,[3] Duangnate Rojanaporn,[4] Rangsima Aroonroch,[5] Bhoom Suktitipat,[6,7] Chonticha Saisawang,[8] Saovaros Svasti,[9,10] Suradej Hongeng,[11] Rossukon Kaewkhaw[2]*

[1]Research Center, Faculty of Medicine Ramathibodi Hospital, Mahidol University, Bangkok, Thailand

[2]Section of Translational Medicine, Faculty of Medicine Ramathibodi Hospital, Mahidol University, Bangkok, Thailand

[3]Lymphocyte cell biology section, National Inst of Arthritis and Musculoskeletal and Skin Diseases (NIAMS), National Institutes of Health (NIH), Bethesda, USA.

[4]Department of Ophthalmology, Faculty of Medicine Ramathibodi Hospital, Mahidol University, Bangkok, Thailand

[5]Department of Pathology, Faculty of Medicine Ramathibodi Hospital, Mahidol University, Bangkok, Thailand

[6]Department of Biochemistry, Faculty of Medicine Siriraj Hospital, Mahidol University Bangkok, Thailand

[7]Integrative Computational BioScience Center, Mahidol University, Nakhon Pathom, Thailand

[8]Institute of Molecular Biosciences, Mahidol University, Nakhon Pathom, Thailand

[9]Department of Biochemistry, Faculty of Science, Mahidol University, Bangkok, Thailand

[10]Thalassemia Research Center, Institute of Molecular Biosciences, Mahidol University, Nakhon Pathom, Thailand

[11]Department of Pediatrics, Faculty of Medicine Ramathibodi Hospital, Mahidol University, Bangkok, Thailand

Correspondence: Rossukon Kaewkhaw, Section of Translational Medicine, Faculty of Medicine Ramathibodi Hospital, Mahidol University, Bangkok, 10400 Thailand. rossukon.kae@mahidol.edu

Supplementary Methods

Genomic Analysis

Genomic DNA was extracted from primary retinoblastoma, blood, and cells using the DNeasy Blood and Tissue Kit (Qiagen, Valencia, CA, USA). MYCN copy number was determined by digital droplet PCR (ddPCR). A 20-μL reaction contained 1× QX200 ddPCR EvaGreen (BioRad Laboratories, Hercules, CA, USA), 50 nM forward primer (I2_MYCN_F) and 100 nM reverse primer (I2_MYCN_R) for MYCN amplicon, 150 nM forward primer (RPP30_F) and 100 nM reverse primer (RPP30_R) for reference gene amplicon (RPP30), and 3 ng of HaeIII-digested DNA template. The droplets were generated by QX200 Droplet Generator (BioRad Laboratories), followed by PCR. Thermal cycling condition consisted of an activation period (95°C for 5 minutes) followed by 40 cycles of two-step thermal profile (95°C for 30 s followed by 60°C for 1 minute), a dye-stabilization step (4°C for 5 minutes, then 90°C for 5 minutes), and finally a 4°C indefinite hold. A 2°C/s ramp rate was applied for all thermal cycling steps. A signal in droplets (16,000–18,000 droplets) was read using the QX200 Droplet Reader (BioRad Laboratories), and copy number was analyzed by QuantaSoft analysis software (BioRad Laboratories). The assay was repeated using *RLBP* as a reference gene, and DNA from healthy controls was included in assays. Primer sequences are listed in Supplementary Table 2.

Whole-genome analysis of tumor and paired blood DNA was performed for patient RB170. The samples were prepared according to the Illumina TruSeq Nano DNA library preparation guide (Illumina Inc., San Diego, CA, USA). The libraries were sequenced using an Illumina HiSeq X sequencer; the read length and throughput mean depth were 150 bp and 45×, respectively. Single nucleotide variants and indels, copy number variants, and structure variants were called using GATK4 Mutect2 [1]. The effects of single nucleotide variants and indels and structure variants were determined using Variant Effect Predictor [2] (VEP; version 90; https://asia.ensembl.org/info/docs/tools/vep/). Variants with population allele frequency <1% in any populations from 1000 Genome Phase 3 (https://www.internationalgenome.org/category/phase-3/), NHLBI-ESP (https://esp.gs.washington.edu/drupal/), gnomAD [3], and TwinsUK data were selected. The

impact of non-synonymous variants was predicted using metaSVM [4], metaLR, FATHMM [5], SIFT [6], and PolyPhen2 [7].

Germline variants were called using Isaac Variant Caller [8] followed by SnpEff [9] for annotation and effect prediction for single-nucleotide variants and small indels, and Manta [10] for structural variants. Copy number alterations and loss of heterozygosity were determined in blood and tumor DNA by using the CGH/SNP array (Infinium CytoSNP-850K array, Illumina Inc.) in accordance with the manufacturer's instructions; the results were analyzed and visualized using Nexus copy number software (BioDiscovery, El Segundo, CA, USA). Multiplex ligation-dependent probe amplification (MLPA) and Sanger sequencing were conducted according to the previous work [11] to determine genetic abnormalities in the *RB1* gene.

RNA Expression Analysis

RNA was extracted from primary retinoblastoma and cells using TRI Reagent (Molecular Research Center Inc., Cincinnati, OH, USA). cDNA was synthesized from 1 µg of total RNA using random primer and ImProm-II Reverse Transcriptase (Promega, Madison, WI, USA). RT-PCR reactions were conducted to amplify *MYCNOS* (variants 1-5)**,** *MYCN*, and coding *RB1* regions. The RT-PCR products of *RB1* were purified using a gel extraction kit (Qiagen) for sequencing. RT-qPCR was performed using iTaq Universal SYBR Green Supermix (BioRad Laboratories) to amplify *MYCN, MYCNOS1,* and *MYCNOS2*. β-Actin (*ACTB*) expression was used to normalize the expression of target genes. Thermal cycling condition of RT-PCR for all variants of MYCNOS consisted of initial denaturation (95°C for 30 s) followed by 30 cycles of three-step thermal profile (95°C for 15 s, 55°C for 30 s, and 68°C for 30 s), and then a final extension step (68°C for 5 minutes), and finally a 4°C indefinite hold. The thermal cycling conditions of RT-qPCR for *MYCN*, *MYCNOS1*, and *MYCNOS2* consisted of initial denaturation (95°C for 20 s) followed by 40 cycles of two-step thermal profile (95°C for 5 s and 60°C for 30 s), followed by melting curve analysis. Thermal cycling

condition of RT-PCR for *RB1* consisted of initial denaturation (98°C for 30 s) followed by 30 cycles of three-step thermal profile (98°C for 10 s, 68°C for 30 s [fragment 1]; 63°C for 30 s [fragment 2]; 60°C for 30 s [fragment 3], and 72°C for 40 s [fragments 1 and 2] or 55 s [fragment 3]), a final extension step (72°C for 5 minutes), and finally a 4°C indefinite hold. Primers are listed in Supplementary Table 2 for *MYCNOS1* to *5*, *MYCN*, *RB1*, and *ACTB* amplification.

Western blotting

Cells were lysed using radioimmunoprecipitation assay (RIPA) lysis and extraction buffer containing halt protease inhibitor cocktail (Thermo Fisher Scientific, Waltham, MA, USA) in accordance with the manufacturer's instructions. Lysates were mixed with Laemmli buffer and freshly added β-mercaptoethanol and boiled at 95°C for 15 minutes. Total proteins were separated on 10% SDS polyacrylamide gel and transferred to the PVDF membrane, which was then incubated in 5% non-fat skim milk and 0.2% Tween in Tris-buffered saline. MYCN protein was probed by overnight incubation with mouse anti-MYCN antibody (1:500 or 1:200, # sc-56729, Santa Cruz Biotechnology, Santa Cruz, CA, USA) at 4°C, followed by horseradish peroxidase (HRP)-conjugated anti-mouse antibody (1:5000, #7076; Cell Signaling Technology, Danvers, MA, USA) and HRP-conjugated rabbit anti-actin antibody (1:5000, #12620; Cell Signaling Technology). Actin protein was used as a loading control. Protein signals were developed using chemiluminescent HRP substrate, in accordance with the manufacturer's instructions, and recorded using the Chemidoc MP Imaging System (Bio-Rad Laboratories).

Protein Stability Assay

Retinoblastoma cells were treated with 10 µg/mL cycloheximide for 0, 15, 30, 60, and 120 minutes before cell lysates were collected. A total protein of 40 µg was used for immunoblotting for MYCN, and the levels of actin were used as a loading control.

Histology, Immunofluorescence, and Imaging

Histology, immunofluorescence, including antibody dilutions, and imaging were conducted according to a previous report [12]. Fluorescent images were acquired by confocal laser scanning microscopy, and Z-stacking was performed with NIS-Element AR (Nikon, Tokyo, Japan).

Soft Agar Colony Formation Assay

Colony formation assays were performed using initial seeding of 0.5 and 1×10^3 cells for Y79 and 1 and 5×10^5 cells for RB170 mixed with 0.36% low-melting temperature agarose solution in growth medium in 6-well plates. The cell-agarose solution mixtures were plated on the solidified bottom layer of 0.75% low-melting temperature agarose in growth medium and grown at 37°C under 5% CO_2 for 3 weeks. Green fluorescent protein (GFP)-positive colonies were visualized, and photographs were recorded using the Operretta high-content imaging system (Perkin Elmer, Waltham, MA, USA). In photographs, a group of GFP-positive colonies larger than 1×10^3 μm^2 (50 cells) was considered a colony, and the size and number of colonies were analyzed using Columbus Image Data Storage and Analysis System (Perkin Elmer). Colonies were stained with 0.05% crystal violet-40% methanol in PBS and photographed by Chemidoc Imaging System (BioRad Laboratories).

Cell Viability Assay and Drug Test

Cell proliferation analysis was performed by measurement of ATP production using the CellTiter-Glo (Promega) luminescent assay in accordance with the manufacturer’s instructions. The produced luminescence was proportional to the number of viable cells in 0-, 1-, 3-, 5-, and 7-day cultures and was read using the Infinite 200 PRO multimode reader (Tecan, Mannedorf, Switzerland). Cells were incubated with carboplatin or topotecan diluted at different concentrations (0, 2, 4, 8, 16, 32, 64, and 128 µM for carboplatin or 0, 7.5, 15,

30, 60,120, 240, 480, 960, and 1920 nM for topotecan) for 48 hours, and the number of viable cells was determined using CellTiter-Glo luminescent assay. The half-maximal effective concentration (EC_{50}) of each drug was calculated from the plots between concentrations and percent of cell viability.

Migration assay

Cells (3×10^5 cells) were grown in the upper chamber of a 24-well Transwell containing Neurobasal medium, while the lower chamber contained Neurobasal medium or full growth medium developed previously [12] with additional epidermal growth factor (EGF, 200 pg/mL) as chemoattractant. Transwell migration assays were conducted for 72 hours in a humidified incubator at 37°C in a 5% CO_2 atmosphere. Cell were fixed and stained with 0.05% crystal violet (w/v) in 40% methanol for 20 minutes. Transwell inserts were washed with distilled water, and cells in the upper chambers were removed using a cotton-tip. The number of migrating cells was counted using an inverted microscope.

Cell Cycle Analysis

Cell cycle was analyzed using FITC BrdU Flow Kit (BD Biosciences/Thermo Fisher, Waltham, MA, USA) in accordance with the manufacturer’s instructions. Briefly, cells were labeled with 10 µM BrdU for 60 minutes before TrypLE-mediated dissociation. Cells were fixed, permeabilized, and stained using fluorescein isothiocyanate (FITC) anti-BrdU antibody for 20 minutes. Cell suspensions were washed and incubated with 7-aminoactinomycin D (7-AAD) solution for 20 minutes. Stained cells were immediately analyzed by flow cytometry. The data were analyzed using FlowJo software (BD Biosciences).

Live Cell Imaging

In total, 5×10^4 cells were seeded on a Hi-Q4 culture dish secured in a chamber maintained at 37°C under a humidified atmosphere of 5% CO_2. Growth medium contained 5% Matrigel

solution to facilitate cell adhesion. Cell were monitored using a Biostation IM_Q (Nikon) equipped with a camera for video recording. Time-lapse video was conducted for 120 hours, and images were captured every 3 hours. Cell motility including velocity magnitude and speed was analyzed using the image analysis software CL-Quant Ver. 2.0 (Nikon).

RNA-sequencing

RNA was extracted from cultures by using TriPure isolation reagent (Roche Applied Science, Penzberg, Germany). The quality and quantity of RNA were determined by RNA6000 assay (Agilent Technologies, Santa Clara, CA, USA). Specimens with an RNA integrity number (RIN) >8.0 were used in this study. RNA Libraries were constructed by using the TruSeq Stranded mRNA LT Sample Prep Kit (Illumina Inc.), in accordance with the manufacturer's instructions. RNA sequencing was performed with the Illumina NovaSeq sequencing system (100-bp paired-end reads); an average of 38 million read counts were generated for each sample.

Processing RNA-seq Reads:

Low-quality reads were purged using Trimmomatic (v0.36) [13]. Quantification of the transcriptome was carried out using Kallisto (v0.43) [14] using annotation from Ensembl version 84 at the transcript level. Gene level counts were generated using the tximport R package [15], and gene count level data were normalized using the TMM (trimmed mean of M-values) method followed by CPM (counts per million) computation.

Differential Expression Analysis:

Differential expression analysis between distinct sample groups was performed using exactTest function as implemented in the edgeR R package [16]. Genes were selected based on a $\log_2$ fold-change value ≥1 and adjusted p-value ≤0.01.

Gene Ontology Analysis:

Gene ontology (GO) analysis for differentially expressed genes was performed using enrichGO function in clusterProfiler R package (v3.6.0) [17]. REVIGO was used to visualize significantly enriched terms (FDR adjusted p-value ≤0.05) [18].

Statistical Analysis

Pearson correlation test was conducted between *MYCN* copy number and expression of *MYCN* or *MYCNOS1*, and *MYCN* and *MYCNOS1* expression. Student's *t*-test was used to test the difference in stability of MYCN protein and drug response following MYCNOS silencing. One-way ANOVA followed by Tukey's multiple comparison test was used to test the difference in expression of RNA and protein, cell cycle phase, caspase 3-positive cells, colony size and number, and migration following MYCNOS silencing. The *F*-test was used for analysis of proliferation curves. When p-value was < 0.05, the results were concluded to be statistically significant. Statistical analyses were conducted using R-packages.

Data Availability

Raw data of RNA-seq and whole genome sequencing data have been deposited in Gene Expression Omnibus (GEO) and Sequence Read Archive (SRA) through accession numbers GSE161449 and PRJNA678350, respectively.

Supplementary Figure 1

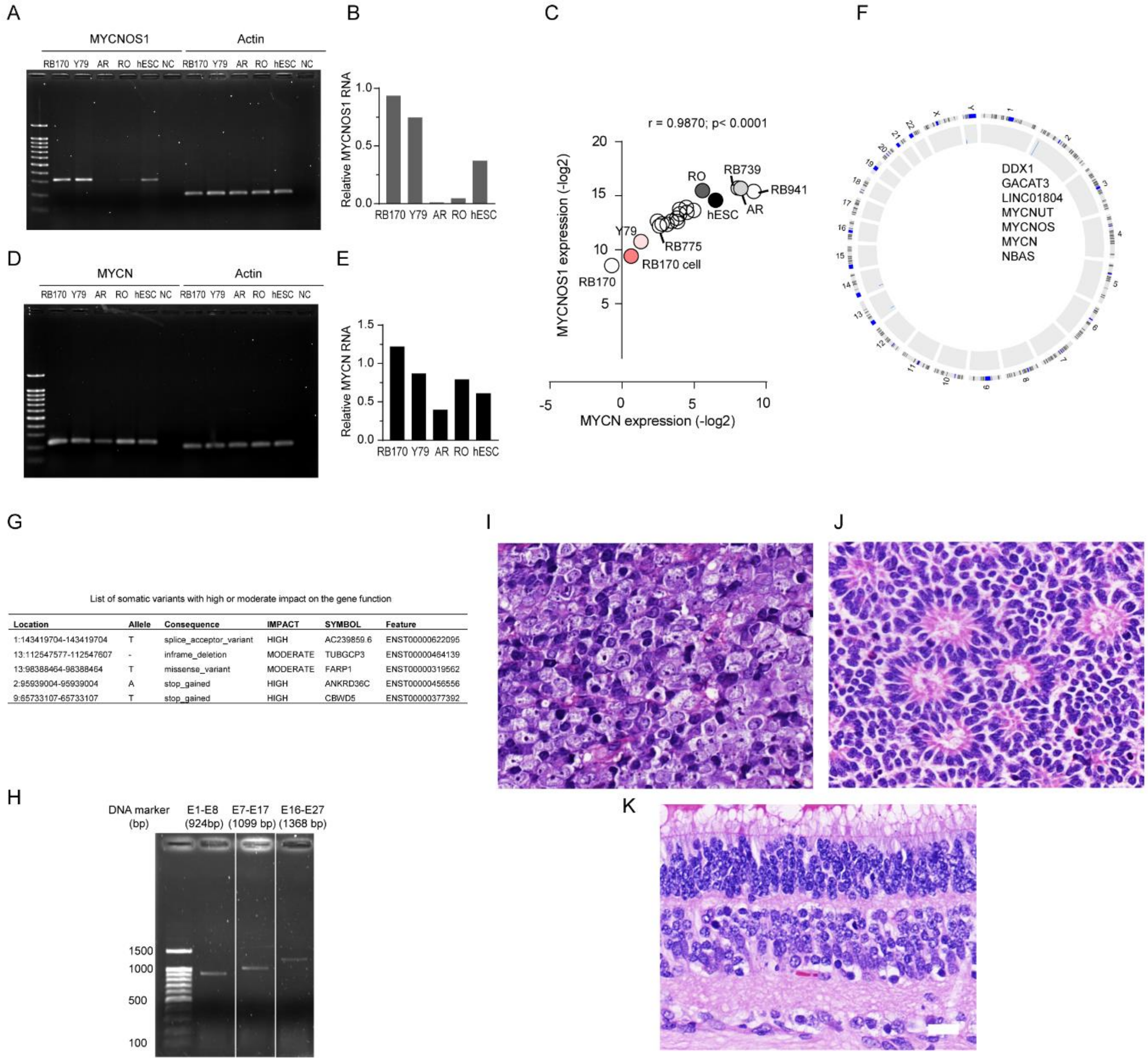

List of somatic variants with high or moderate impact on the gene function

Location	Allele	Consequence	IMPACT	SYMBOL	Feature
1:143419704-143419704	T	splice_acceptor_variant	HIGH	AC239859.6	ENST00000622095
13:112547577-112547607	-	inframe_deletion	MODERATE	TUBGCP3	ENST00000464139
13:98388464-98388464	T	missense_variant	MODERATE	FARP1	ENST00000319562
2:95939004-95939004	A	stop_gained	HIGH	ANKRD36C	ENST00000456556
9:65733107-65733107	T	stop_gained	HIGH	CBWD5	ENST00000377392

Fig. S1. Molecular and histological features of *MYCN*-amplified retinoblastoma. (**A**, **B**) Expression levels and densitometry of *MYCNOS1* by RT-PCR in RB170 tumor tissue compared with Y79, adult retina (AR), human embryonic stem cell (hESC)-derived retinal organoid (RO) representative of fetal retina, and hESC. The negative control (NC) is the reaction without cDNA. (**C**) Correlation of *MYCNOS1* with MYCN expression examined by RT-qPCR. Correlation analysis was conducted using Pearson correlation method in which correlation coefficient (r) was computed. When p-value was < 0.05, two variables were significantly correlated. (**D**, **E**) Expression levels and densitometry of *MYCN* by RT-PCR. (**F**, **G**) Somatic mutation analysis of RB170 tumor tissue showing high *MYCN* amplification and

co-amplified genes (**F**) and mutations detected in five genes (**G**). (**H**) *RB1* transcripts in RB170 tumor tissue by RT-PCR. PCR products were extracted and purified for sequencing. (**I**–**K**) Hematoxylin and eosin (H&E) staining showing blast cells with prominent nucleoli of RB170 tumor tissue (**I**) compared with retinoblastoma with typical rosettes (**J**) and adjacent normal retina (**K**). Scale bar: 20 µm.

Supplementary Figure 2

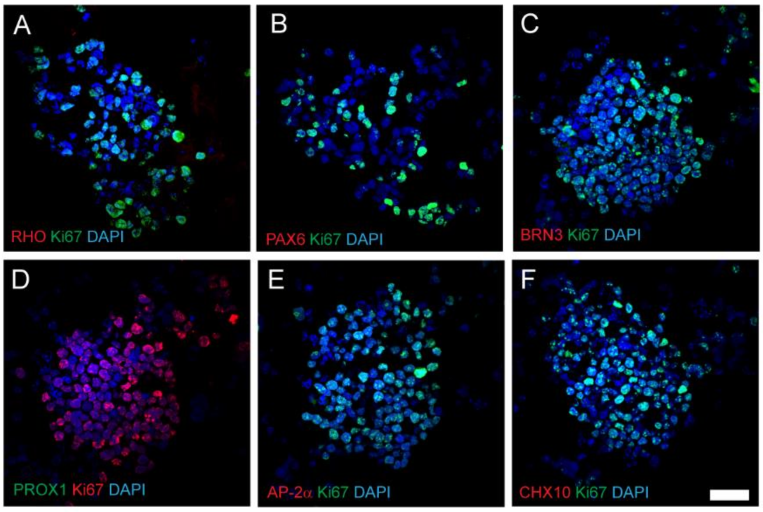

Fig. S2. Immunostaining of RB170 tumor cells. (**A**–**F**) Immunostaining indicative of rod (RHO), ganglion (PAX6 and BRN3), amacrine (PROX1, AP2-α, and PAX6), horizontal (PROX1 and PAX6), bipolar (CHX10), and retinal progenitor (CHX10 and PAX6) cells. The retinal markers were co-stained with Ki67, indicative of proliferation.

Supplementary Figure 3

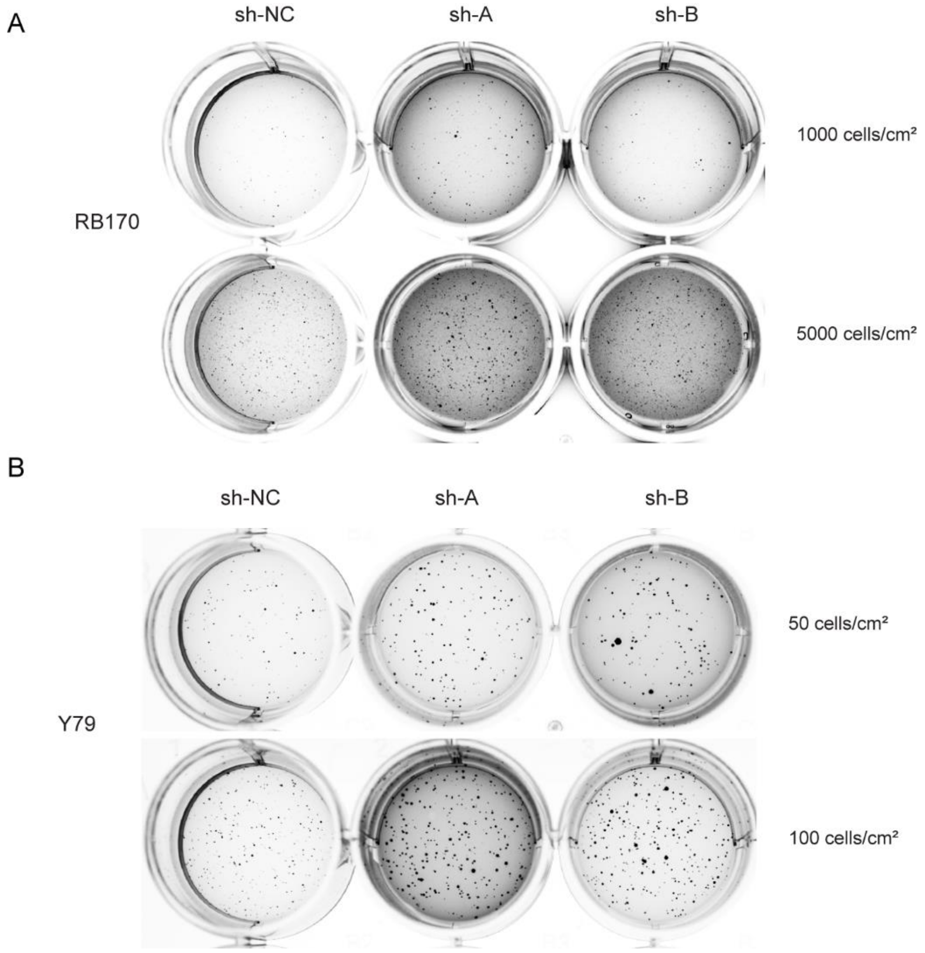

Fig. S3. Soft agar colony formation assay. (**A**, **B**) Colony forming assay with initial seeding densities at 1000 and 5000 cells/cm^2 for RB170 (**A**) and 50 and 100 cells/cm^2 for Y79 (**B**).

Supplementary Figure 4

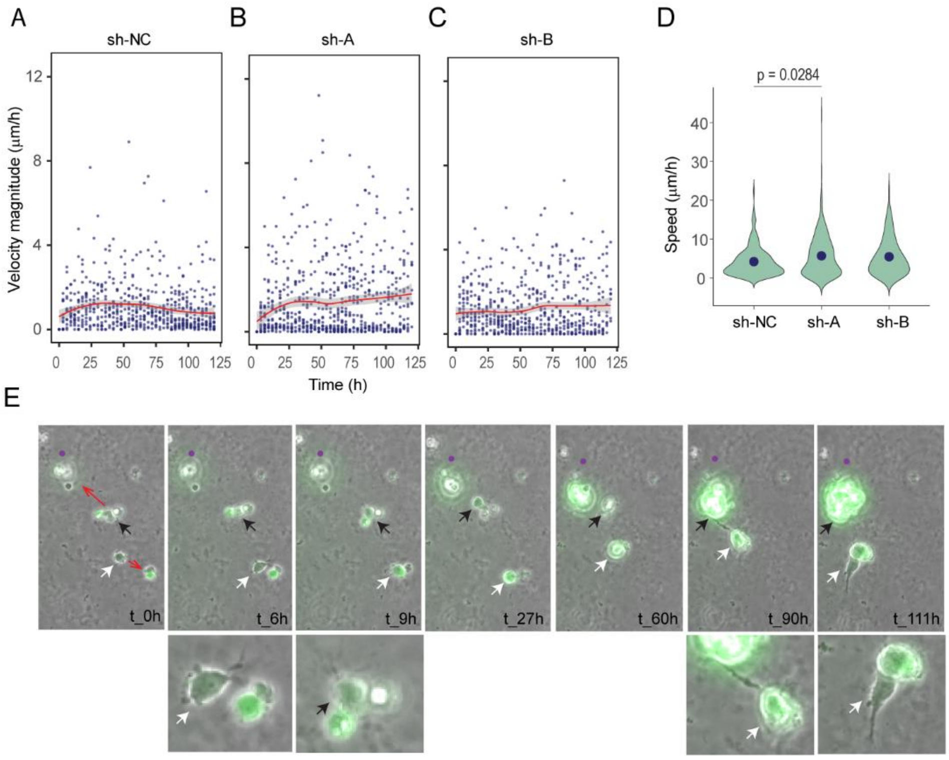

Fig. S4. Reduced *MYCNOS1* expression associated with cell motility. (**A**–**D**) Velocity magnitude (**A**–**C**) and speed (**D**) of RB170 deficient for *MYCNOS1* by time-lapse video microscopy over the course of 125 hours, with images taken every 3 hours. (**E**) Snapshots of RB170 cells deficient for *MYCNOS1* forming dendrites and exhibiting more motility (see Supplementary Video). Black and white arrows indicated moving cells toward others, while red arrow pointed out the direction of movement.

Supplementary Figure 5

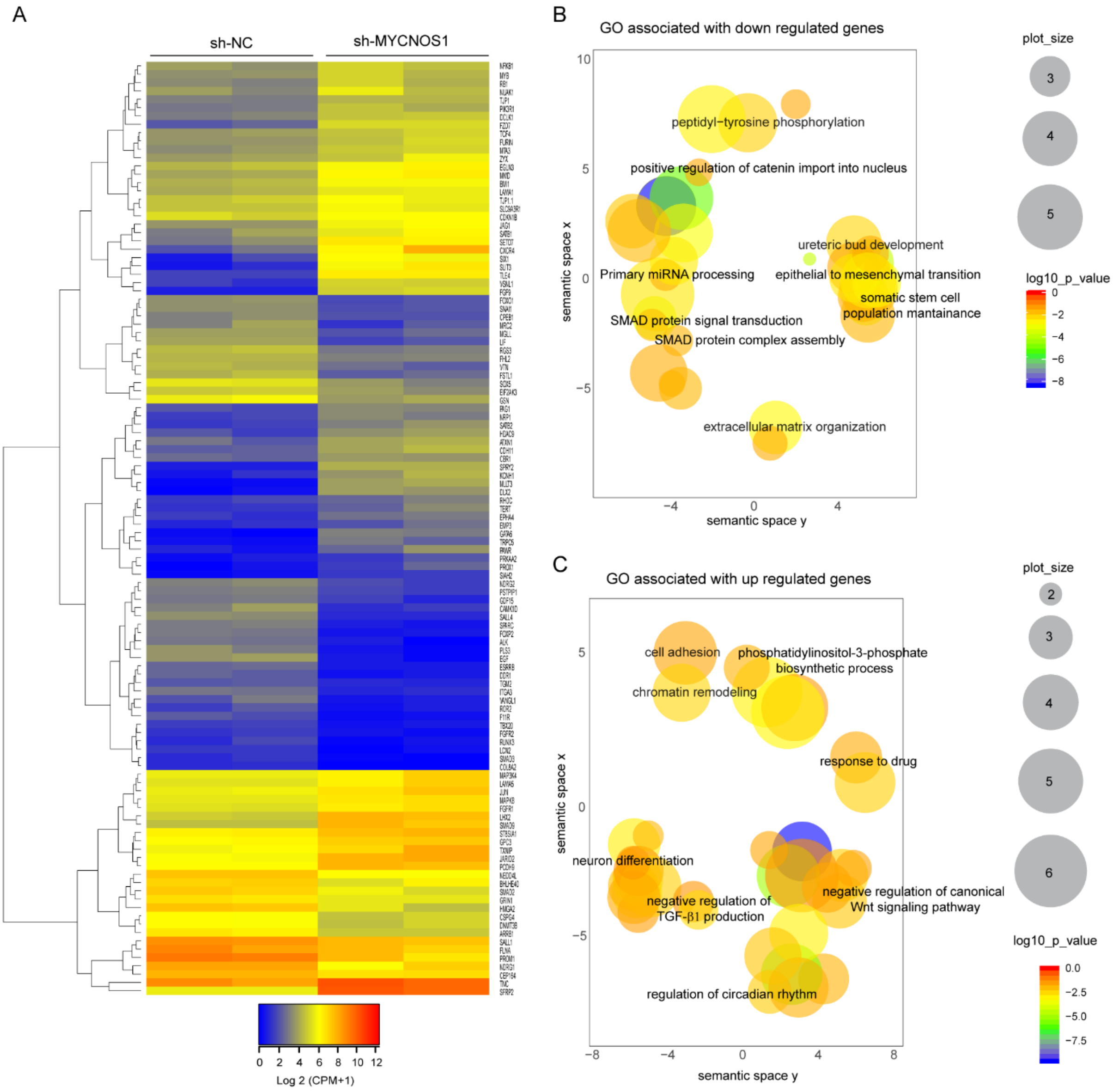

Fig. S5. Analysis of genes associated with epithelial-mesenchymal transition (EMT). (**A**) EMT-associated genes (112 genes; 49 downregulated and 63 upregulated) in RB170 deficient for *MYCNOS1*. (**B**, **C**) Gene ontology analysis of downregulated (**B**) and upregulated (**C**) genes associated with EMT in RB170 deficient for *MYCNOS1* compared with RB170 with short hairpin (sh)-NC.

Supplementary Video

Time-lapse video microscopy over the course of 120 hours with images captured every 3 hours**.**

Supplementary Table 1. Short hairpin RNA sequence for *MYCNOS1* transcript

Short hairpin RNA	Sequence (5′-3′)
Sh-A	AAATACATTTCTTTTCTTTA
Sh-B	CCTGGCACTTCAAGAGTCTGA

Supplementary Table 2. List of primers

Gene	Primer name		Primer sequence (5′-3′)	Amplicon size (bp)	Remark
MYCNOS	E2_MYCNOS_F<br>E4_MYCNOS1_R	Variant 1	GCTCTCCGACAGCTCAAAC<br>GGCCTTCATGGCGTACCATC	328	RT-PCR*
	E1_MYCNOS2_F<br>E3_MYCNOS_R	Variant 2	TCACGAGCACGCAGACAAC<br>GACAAGCAATTGCCAGGCTC	361	
	E1_MYCNOS3_F<br>E3_MYCNOS_R	Variant 3	GCAGCAGCTCAAACTTCTTCC<br>GACAAGCAATTGCCAGGCTC	353	
	E2_MYCNOS4_F<br>E3_MYCNOS_R	Variant 4	AGCAGCCGTGACCCAGATTG<br>GACAAGCAATTGCCAGGCTC	340	
	E2_MYCNOS_F<br>E3_MYCNOS5_R	Variant 5	GGCTCTCCGACAGCTCAAAC<br>CCTCTAGACCCCAGTAGGAGTG	327	
	E3_MYCNOS1_F1<br>E4_MYCNOS1_R	Variant 1	CGTGACACTGTGGAAGACAGGC<br>GGCCTTCATGGCGTACCATC	126	RT-qPCR†
	MYCNOS_E2_F<br>MYCNOS_E3_R	Variant 2	GGCTCTCCGACAGCTCAAAC<br>GACAAGCAATTGCCAGGCTC	226	
RB1	E1_F<br>E8_R	Fragment 1	TCCGGTTTTTCTCAGGGGACG<br>CCGTGCACTCCTGTTCTGACC	924	RT-PCR
	E7_F<br>E17_R	Fragment 2	GCTAAAGGGGAAGTATTACAAATGG<br>AATCTGAGAGCCATGCAAGG	1099	
	E16_F<br>E27_R	Fragment 3	AGCAAACTTCTGAATGACAAC<br>TCATTTCTCTTCCTTGTTTGAGG	1368	
MYCN	MYCN_I2_F<br>MYCN_I2_R		CCAAAGAGTGGCATTGCCTTG<br>CCTCACCAAAAGCTGTCAAGGC	219	ddPCR#
	MYCN_E2_F<br>MYCN_E3_R		ACCACAAGGCCCTCAGTAC<br>TCGTTTGAGGATCAGCTCGC	224	RT-PCR/RT-qPCR
ACTB	Human B-actin_F<br>Human B-actin_R		GGCACCCAGCACAATGAAGATC<br>GTAACGCAACTAAGTCATAGTCCGC	195	RT-PCR/<br>RT-qPCR
RLBP1	RLBP1_F<br>RLBP1_R		GTGAATTTCCGGCTGCAGTACC<br>ACTAGAGAGGACACCAGGGTAG	99	ddPCR
RPP30	RPP30_F<br>RPP30_R		AAGAGGCTTGCTGTTTGGGC<br>TTACCTCCATGGAGAAGCGC	86	ddPCR

*RT-PCR: reverse transcription PCR; †RT-qPCR: quantitative reverse transcription PCR; #ddPCR: droplet digital PCR

References

1. Cibulskis K, Lawrence MS, Carter SL, et al. Sensitive detection of somatic point mutations in impure and heterogeneous cancer samples. *Nat Biotechnol* 2013;31:213-219.
2. McLaren W, Gil L, Hunt SE, et al. The Ensembl Variant Effect Predictor. *Genome Biol* 2016;17:122.
3. Whiffin N, Karczewski KJ, Zhang X, et al. Characterising the loss-of-function impact of 5' untranslated region variants in 15,708 individuals. *Nat Commun* 2020;11:2523.
4. Kim S, Jhong JH, Lee J, Koo JY. Meta-analytic support vector machine for integrating multiple omics data. *BioData Min* 2017;10:2.
5. Shihab HA, Gough J, Cooper DN, et al. Predicting the functional, molecular, and phenotypic consequences of amino acid substitutions using Hidden Markov Models. *Hum Mutat* 2013;34:57-65.
6. Ng PC, Henikoff S. SIFT: predicting amino acid changes that affect protein function. *Nucleic Acids Res* 2003;31:3812-3814.
7. Adzhubei I, Jordan DM, Sunyaev SR. Predicting functional effect of human missense mutations using PolyPhen-2. *Curr Protoc Hum Genet* 2013; 76:7.20.1-7.20.41.
8. Raczy C, Petrovski R, Saunders CT, et al. Isaac: ultra-fast whole-genome secondary analysis on Illumina sequencing platforms. *Bioinformatics* 2013;29:2041-2043.
9. Cingolani P, Platts A, Wang LL, et al. A program for annotating and predicting the effects of single nucleotide polymorphisms, SnpEff: SNPs in the genome of Drosophila melanogaster strain w1118; iso-2; iso-3. *Fly (Austin)* 2012;6:80-92.
10. Chen XY, Schulz-Trieglaff O, Shaw R, et al. Manta: rapid detection of structural variants and indels for germline and cancer sequencing applications. *Bioinformatics* 2016;32:1220-1222.
11. Rojanaporn D, Boontawon T, Chareonsirisuthigul T, et al. Spectrum of germline RB1 mutations and clinical manifestations in retinoblastoma patients from Thailand. *Mol Vis* 2018;24:778-788.
12. Saengwimol D, Rojanaporn D, Chaitankar V, et al. A three-dimensional organoid model recapitulates tumorigenic aspects and drug responses of advanced human retinoblastoma. *Sci Rep* 2018;8:15664.
13. Bolger AM, Lohse M, Usadel B. Trimmomatic: a flexible trimmer for Illumina sequence data. *Bioinformatics* 2014;30:2114-2120.
14. Bray NL, Pimentel H, Melsted P, Pachter L. Near-optimal probabilistic RNA-seq quantification. *Nat Biotechnol* 2016;34:525-527.
15. Soneson C, Love MI, Robinson MD. Differential analyses for RNA-seq: transcript-level estimates improve gene-level inferences. *F1000Res* 2015;4:1521.
16. Robinson MD, McCarthy DJ, Smyth GK. edgeR: a Bioconductor package for differential expression analysis of digital gene expression data. *Bioinformatics* 2010;26:139-140.
17. Yu GC, Wang LG, Han YY, He QY. clusterProfiler: an R package for comparing biological themes among gene clusters. *OMICS* 2012;16:284-287.
18. Supek F, Bošnjak M, Škunca N, Šmuc T. REVIGO summarizes and visualizes long lists of gene ontology terms. *PLoS One* 2011;6:e21800.
